# Potential biomarkers of DNA replication stress in cancer

**DOI:** 10.18632/oncotarget.16940

**Published:** 2017-04-07

**Authors:** Liqun Ren, Long Chen, Wei Wu, Lorenza Garribba, Huanna Tian, Zihui Liu, Ivan Vogel, Chunhui Li, Ian D. Hickson, Ying Liu

**Affiliations:** ^1^ Basic Medical Research Institute, Chengde Medical University, Chengde, China; ^2^ Center for Chromosome Stability, Department of Cellular and Molecular Medicine, University of Copenhagen, Copenhagen, Denmark; ^3^ Pathology Department, Affiliated Hospital, Chengde Medical University, Chengde, China; ^4^ Center for Healthy Aging, Department of Cellular and Molecular Medicine, University of Copenhagen, Copenhagen, Denmark

**Keywords:** cancer biomarker, chromosome instability, common fragile sites, MiDAS, replication stress, Chromosome Section

## Abstract

Oncogene activation is an established driver of tumorigenesis. An apparently inevitable consequence of oncogene activation is the generation of DNA replication stress (RS), a feature common to most cancer cells. RS, in turn, is a causal factor in the development of chromosome instability (CIN), a near universal feature of solid tumors. It is likely that CIN and RS are mutually reinforcing drivers that not only accelerate tumorigenesis, but also permit cancer cells to adapt to diverse and hostile environments. This article reviews the genetic changes present in cancer cells that influence oncogene-induced RS and CIN, with a particular emphasis on regions of the human genome that show enhanced sensitivity to the destabilizing effects of RS, such as common fragile sites. Because RS exists in a wide range of cancer types, we propose that the proteins involved counteracting this stress are potential biomarkers for indicating the degree of RS in cancer specimens. To test this hypothesis, we conducted a pilot study to validate whether some of proteins that are known from *in vitro* studies to play an essential role in the RS pathway could be suitable as a biomarker. Our results indicated that this is possible. With this review and pilot study, we aim to accelerate the development of a biomarker for analysis of RS in tumor biopsy specimens, which could ultimately help to stratify patients for different forms of therapy such as the RS inhibitors already undergoing clinical trials.

## INTRODUCTION

Despite huge advances in our understanding of basic cancer biology, the incidence of cancer worldwide is still rising, and the overall survival rates for patients with advanced cancer are still low. For example, a World Health Organization report in 2012 revealed that there were approximately 14 million newly recorded cases of cancer worldwide that year, as well as over 8 million cancer-related deaths [1]. Four years on from this report, the same general picture remains, with a particularly negative outlook existing for advanced cancers. Taking colon cancer cases recorded during 2014-2015 in the USA as an example, those patients with early stage disease (Stage I) had a greater than 90% chance of surviving for more than 5 years after diagnosis. Unfortunately, however, only around 40% of cases are diagnosed at such an early disease stage. In those cases where the cancer had spread to distant organs (stage IV patient), the 5-year survival dropped dramatically to only around 15%. The five-year survival rate for all colon cancer is currently around 65% [2]. Clearly, to significantly improve the survival of cancer patients, progress must be made in targeting those cancers that are at an advanced stage at the point of diagnosis.

Cancer cells differ from most normal cells in that they display an unlimited capacity for proliferation. This altered proliferative capacity is a consequence of the effects of genetic changes that arise during tumorigenesis, which are generally acquired as part of a sequential, multi-step process. Somewhat ironically, the same mutation-selection evolutionary law that Darwin described for the development of different species shapes this pathological ‘evolutionary’ process. In the case of cancer development, the mutations are either inherited, or more frequently acquired somatically. Recent cancer genome sequencing efforts have revealed a bewildering array of different mutations in many of the most common cancers. Nevertheless, in many cases, there appears to be a common set of genes mutated characteristically in a particular tumor type. Cells carrying these genetic changes are then subjected to powerful selection imposed by the environment in which the cell resides. As a result, cancers often ‘evolve’ differently in different organs and even in the same organ from different patients. Moreover, their ability to invade surrounding tissue, as well as to spread to and survive within distant organs, can be very different. This genetic and phenotypic diversity has imposed huge challenges for the development of curative therapies because ‘one size clearly does not fit all’ when it comes to selection of the appropriate treatment options.

It is well established that cancers arise primarily as a result of a series of changes to two classes of genes: oncogenes and tumor suppressor genes. These changes result either in the activation of the oncogene or in the inactivation of the tumor suppressor gene, and are often termed ‘driver mutations’. For example, approximately 15 different driver mutations (as well as >60 ‘passenger’ mutations that are not directly involved in tumorigenesis) have been found in colon cancers [3]. When oncogenes become activated, one of the main consequences is the development of so-called ‘DNA replication stress’ (RS). While RS lacks a clear universal definition, it is usually taken to mean a situation in which DNA replication forks are disrupted leading to DNA damage and/or the accumulation of single stranded DNA. This leads to a DNA damage checkpoint response associated with the activation of the ATR and CHK1 kinases. If this disruption to replication is not dealt with promptly or appropriately, RS can lead to detrimental consequences including chromosome translocations or whole chromosome missegregation during mitosis leading to aneuploidy. Although initially considered a rare event associated only with a limited set of oncogenes, recent research has revealed that RS is triggered by the activation of a wide range of oncogenes, including many of the most common cancer-associated events such as c-Myc amplification, mutation of Ras, and overexpression of Cyclin D or the replication licensing factors Cdt1 and Cdc6 [4, 5].

There is a large body of evidence to indicate that chromosomal instability (CIN) in cancer cells is a driving force during tumorigenesis, and one of the major causes not only of heterogeneity in cancer cells, but also of cancer cell resistance to radiotherapy and chemotherapy [6]. As alluded to above, it has been proposed that RS plays a central role in the generation of structural and numerical CIN [7]. Indeed, it is conceivable that CIN and RS are mutually reinforcing drivers that permit cancer cells to have a selective advantage when adapting to a challenging environments. This raises the question of whether we can define a common molecular pathway or process that exists in many, if not all, cancers, that would allow us to identify a useful cancer biomarker? This article will focus on the genetic changes present in cancer cells that influence oncogene-induced RS and CIN, with a particular emphasis on common fragile sites (CFSs) in the human genome because of an accumulating body of evidence indicating that these loci are major hotspots for cancer-associated genome rearrangements due to their exquisite sensitivity to RS. We will also present pilot data pertaining to the identification of potential markers of RS in cancer biopsies. Ultimately, such a marker would help to stratify patients on the basis of whether they might be particularly vulnerable to the acquisition of advanced-stage and/or therapy-resistant disease.

## RESULTS

### Chromosomal changes in cancer cells

**Figure 1:**
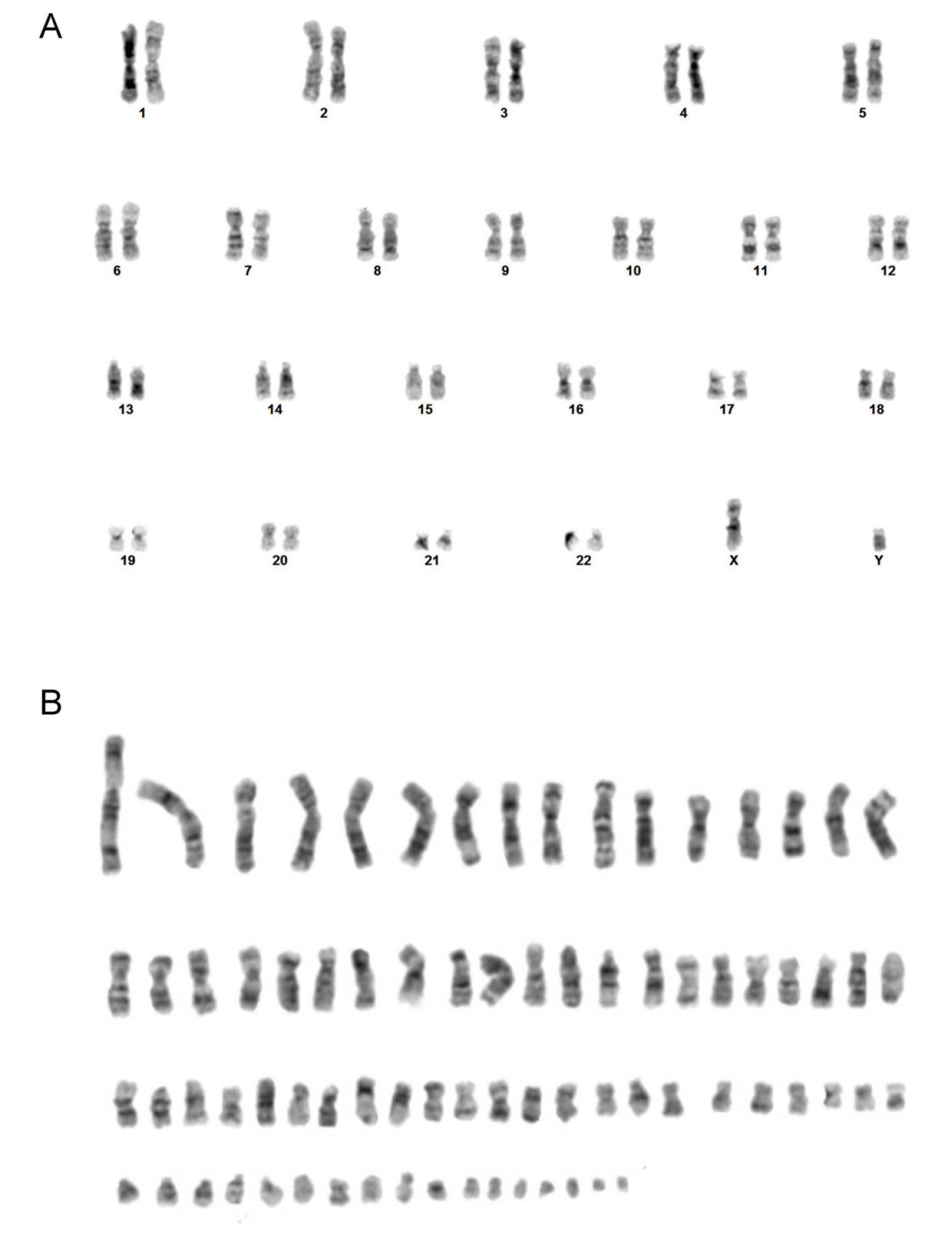
Representative karyotypes of a virally transformed lymphocyte from a normal individual (GM06865) (A), and of an osteosarcoma cell line (U2OS) with CIN (B) Unlike the diploid normal lymphocyte, the aneuploidy U2OS cell karyotype is highly abnormal with many chromosomes showing such an abnormal G-banding pattern that they could not be reliably assigned.

More than 100 years ago, Theodor Boveri speculated on the role of chromosome instability in cancer, based on his work in sea urchins. He reasoned that ‘a cancerous tumor begins with a single cell in which the makeup of its chromosomes becomes scrambled, causing the cells to divide uncontrollably’ [8]. It is now well established that cancer cells display two distinct types of karyotypic abnormalities. The first is a change in chromosome number (aneuploidy), and the second is a change in the structure of individual chromosomes caused by exchanges or rearrangements. These changes can be observed using conventional karyotyping (Figure 1), but are more evident when using a more advanced method such as spectral karyotyping (SKY), which employs fluorescently-labeled probes to mark all of the DNA originating from a particular chromosome. It is known that about 85% of solid tumors show CIN in the form of aneuploidy, which is also the underlying cause of copy number changes of proto-oncogenes [9]. Indeed, we now know that many of the structural changes in individual chromosomes are directly responsible for the activation of oncogenes (via translocation or duplication) or inactivation of tumor suppressor genes (via deletion or mutation).

### Chromosome number changes

The chromosome number changes that are characteristic of cancers are frequently caused by the inaccurate segregation of chromosomes during mitosis. To ensure that an equal number of chromosomes are distributed to the two daughter cells, progression through the key stages of mitosis is monitored by a series of checkpoints involving over 100 proteins. Thus far, four major changes have been described that can lead to the onset of chromosome number changes in tumors. The first is a defect in the spindle assembly checkpoint (SAC) caused by mutation or deregulation of any of several proteins, including *BUB1B*, *MAD1L1*, *MAD2L1*, and CENPE [10–12]. The second cause is a defect in microtubule attachment, with perhaps the most destructive form being so-called ‘merotely’, where one kinetochore becomes attached to microtubules from opposite spindle poles. Kinetochores are often observed to be merotelically attached to spindles in the early stages of mitosis, but this is generally corrected during metaphase [13, 14]. However, when aberrantly attached kinetochores persist until anaphase, sister chromatid missegregation usually results. The control of kinetochore-microtubule attachment is governed by an extensive network of different proteins, including PLK1, the Aurora A and B kinases, and cyclin-CDK complexes, which have been reported dysregulated in cancer cells (reviewed in [15]). A third cause for chromosome number changes is the existence of supernumerary centrosomes in mitotic cells, which can result either directly through a failure to suppress centriole over-duplication [16] or indirectly because of an aborted mitosis due to cytokinesis failure. The fourth major cause is via defects in the cohesin protein complex that holds sister chromatids together after DNA replication until anaphase. For example, loss of function mutations in *STAG2,* which encodes the cohesin protein complex subunit SA-2, has been found in cancer cell lines and in primary tumors [17]. Indeed, CIN can be induced in a karyotypically stable cell line following transfection of a mutated *STAG2* cDNA [17]. Similarly, some CIN cell lines have defects in the recruitment of Shugoshin 1 protein (SGO1) that coordinates sister chromatid cohesion and kinetochore-microtubule attachment [18].

### Chromosome structural changes

Recent cancer genome sequencing efforts have revealed that a set of unstable regions of the genome called common fragile sites (CFSs) lead to recurrent chromosomal structural changes in cancers. This assertion is based on three observations: (i) Some oncogenes or tumor suppressor genes are encoded within CFS loci [19]; (ii) CFS sequences are frequently found at the breakpoints of cancer-specific DNA translocations [20–24], and (iii) A significant proportion of the regions most frequently associated with focal deletions in cancer lie in CFS loci [25]. CFSs are chromosomal loci that tend to form a gap, break or constriction that is visible on condensed metaphase chromosomes. Despite their inherent instability, CFSs are evolutionally conserved chromosomal regions and, for example, generally map to syntenic regions of mouse and human chromosomes [26]. Moreover, they are present in all individuals. Their broken appearance (usually termed CFS ‘expression’) can be induced by exposing cells to agents that partially inhibit DNA synthesis, such as the DNA polymerase inhibitor aphidicolin (APH) [27]. To date, more than 200 CFSs have been identified in the human genome using this method [28].

Great advances have been made towards understanding the mechanism underlying CFS expression. The currently accepted model is that CFS expression reflects an inability to effect chromatin condensation due to a failure to complete DNA replication at the locus prior to mitotic entry [29, 30]. This, of course, begs the question as to why completion of DNA replication would ever be impaired in some regions of the human genome. To address this question, several research groups have sought to define the molecular events leading to CFS expression following replication perturbation. One revealing feature of CFSs is that they recruit several DNA repair and DNA damage response proteins during conditions of RS, including ATR [31], BRCA1 [32], CHK1 [33], FANCD2 [34], RAD51, and γH2AX [35]. In our laboratory, we have focused on the FANCD2 protein, which serves as excellent surrogate marker of the location of CFSs undergoing RS, because it binds to CFS loci irrespective of whether the chromosome is broken or not in metaphase [36]. FANCD2 foci first associate with CFSs in late S/G2 and remain there through mitosis, ultimately segregating evenly with the sister chromatids in anaphase. Indeed, it is clear that under-replicated CFSs recruit multiple DNA repair proteins, including the DNA structure-selective endonuclease MUS81-EME1 in early mitosis, and that this promotes CFS expression [37]. This discovery is consistent with the notion that the expression of CFSs is not an accidental event, but instead is a programmed and regulated process. Indeed, we have proposed that CFS expression is actually beneficial for the maintenance of genome stability, because it prevents much more hazardous events, such as irreversible chromosome missegregation [37].

It has also been demonstrated recently that FANCD2 plays a direct role in CFS replication through an ability to promote the resolution of DNA:RNA hybrids [38]. In the context of CFSs, R-loops likely form due to replication being impeded by the presence of ongoing transcription at the locus. This would likely be particularly problematic in cases of a head-on collision between the replication and transcription machineries. In this context, it is clear that several CFSs have an unusual genomic structure that often incorporates a very long gene [39, 40]. Hence, transcription of the gene takes so long to complete (sometimes more than one entire cell cycle) that a collision between the transcription machinery and the replisome is inevitable at some point during S-phase. Considering that the FANCD2 protein is part of the so-called Fanconi anemia pathway, inactivation of which is responsible for a debilitating disorder associated with a predisposition to various cancers, its association with CFSs provides another line of evidence to suggest that the proper replication of CFSs is crucial for the suppression of oncogenesis.

### Replication stress in cancer cells

In the past decade, evidence has emerged for a positive correlation between tumorigenesis and the degree of RS [41–44]. This suggests that RS is a plausible candidate for being a valuable biomarker in solid tumors. It is therefore essential to obtain detailed knowledge on the events and proteins involved in dealing with this form of stress in human cells. It is clear that, at the molecular level, the RS associated with oncogene activation in cancers can be generated in several ways, such as by atypical DNA secondary structures, including hairpins or G-quadruplexes, a specific chromatin structure that impedes replication fork progression or a collision between the replisome and the transcription machinery operating on the same template. Exogenous stress factors can also generate RS, including several classes of DNA damaging agents, as well as drugs that cause depletion or imbalance of dNTPs (reviewed in [45]). Furthermore, factors required for replication fork stability, repair and restart in response to RS, such as the BRCA1 and BRCA2 proteins, SLX4, and FA group members, have all been shown to associated with cancer development when their function is compromised in either human or mouse models (reviewed in [46]). This indicates that cancer cells have indeed developed various ways to cope with RS.

In normal cells, the ATR-CHK1 kinase cascade is employed to cope with RS (reviewed in [47]. However, the detailed mechanism underlying how stalled replication forks are repaired and DNA synthesis is re-initiated is still largely unknown. Several models, which are not mutually exclusive, have been proposed to explain this process. These include the re-priming of DNA synthesis at a non-origin site, the activation of dormant origins to rescue sites of slow replication, template switching, and homologous recombination-based processes such as break-induced replication (BIR). In cancer cells, there is evidence to show that an abnormal DNA damage response is more likely to be associated with advanced cancer stage, and chemotherapy resistance although the results remains controversial due to the different tissue type and genetic background of the samples analyzed [48–51]. Moreover, there is evidence to indicate that some of the pathways that might permit cells to resist persistent RS are highly activated; for example, the error prone TLS pathway [52, 53]. This suggests that the response to RS in cancer cells is deregulated in a more general way. This, in turn, could lead to CIN when cells containing under-replicated chromosomal regions (e.g. CFS regions) enter mitosis, because these regions have a tendency to missegregate in anaphase. Disturbances such as this in anaphase have the potential to drive chromosome nondisjunction or the formation of micronuclei in daughter cells. Interestingly, micronuclei are now recognized as a source of an extreme form of CIN called chromothripsis (a phenomenon by which multiple chromosomal rearrangements occur in a single event in localized genomic regions in one or a few chromosomes) that is increasingly being detected in cancer genomes.

Recently, we demonstrated that DNA synthesis can still be occurring at CFSs after the cell has entered mitosis, if the cells were treated with a low dose of APH in S phase [54]. We showed that entry into mitotic prophase triggers the recruitment of MUS81-EME1 to CFSs, and the nuclease activity of MUS81 then promotes a form of DNA synthesis that requires POLD3, a non-catalytic subunit of DNA Polymerase. POLD3 is the human ortholog of yeast Pol32, which plays a specialized role in certain forms of DNA repair in yeast, most notably BIR. Indeed, previous studies in human cells have proposed that POLD3 functions in a BIR-like pathway that is responsible for creating segmental genomic duplications [55]. It is most likely that the POLD3-dependent DNA synthesis present in mitosis (which we termed MiDAS; [56]) serves as a ‘rescue’ strategy in the cell to minimize chromosome missegregation and non-disjunction at CFSs in mitosis. Indeed, our data indicated that MiDAS play a particularly important role in the survival of cancer cells with CIN [54]. Very recently, we have demonstrated further that MiDAS often involves DNA synthesis taking place on a single sister chromatid, which is a hallmark of BIR [56].

Clearly, there is a need to define a set of markers that can indicate RS levels or the types of response to RS in cancer cells, which can be used for diagnosis, prognosis or for influencing therapeutic strategies for cancer. Despite the fact that inhibitors of the ATR and CHK1 kinases are undergoing clinical trials (reviewed in [57]), a biomarker for reliably indicating the level of RS is still not available for use in the clinic. The ideal biomarker assay for properly ‘staging’ a cancer patient should be sensitive, rapid, cost-effective, and robust against inter-operator and inter-institutional variability. In this regard, it has been proven very challenging to translate the information obtained from the laboratory to the ‘bedside’ (reviewed in [58]).

### A pilot study of possible markers on RS response

**Figure 2:**
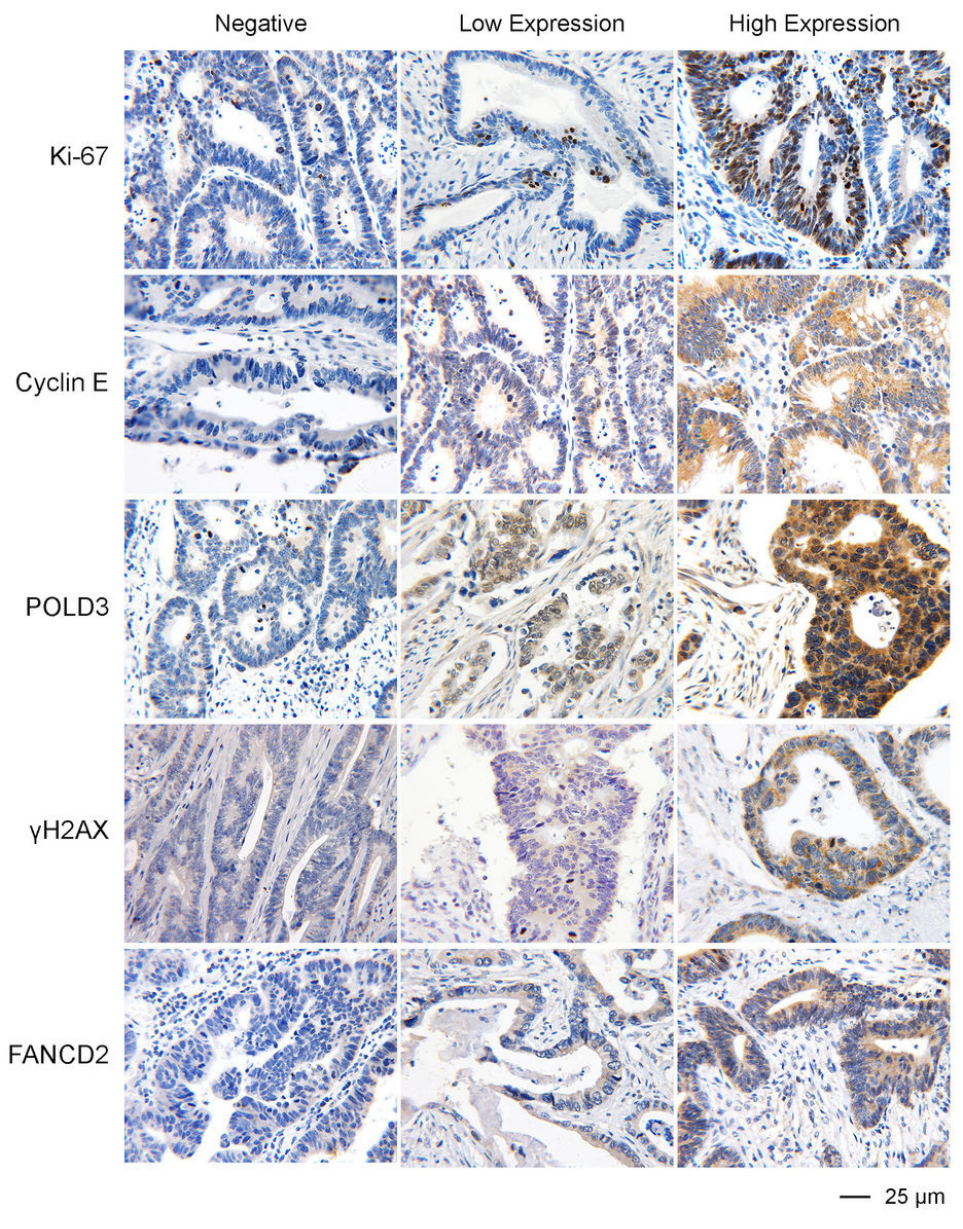
Representative IHC images of the expression of Ki-67, Cyclin E, POLD3, γH2AX, and FANCD2 in colon cancer FFPE specimens

**Figure 3:**
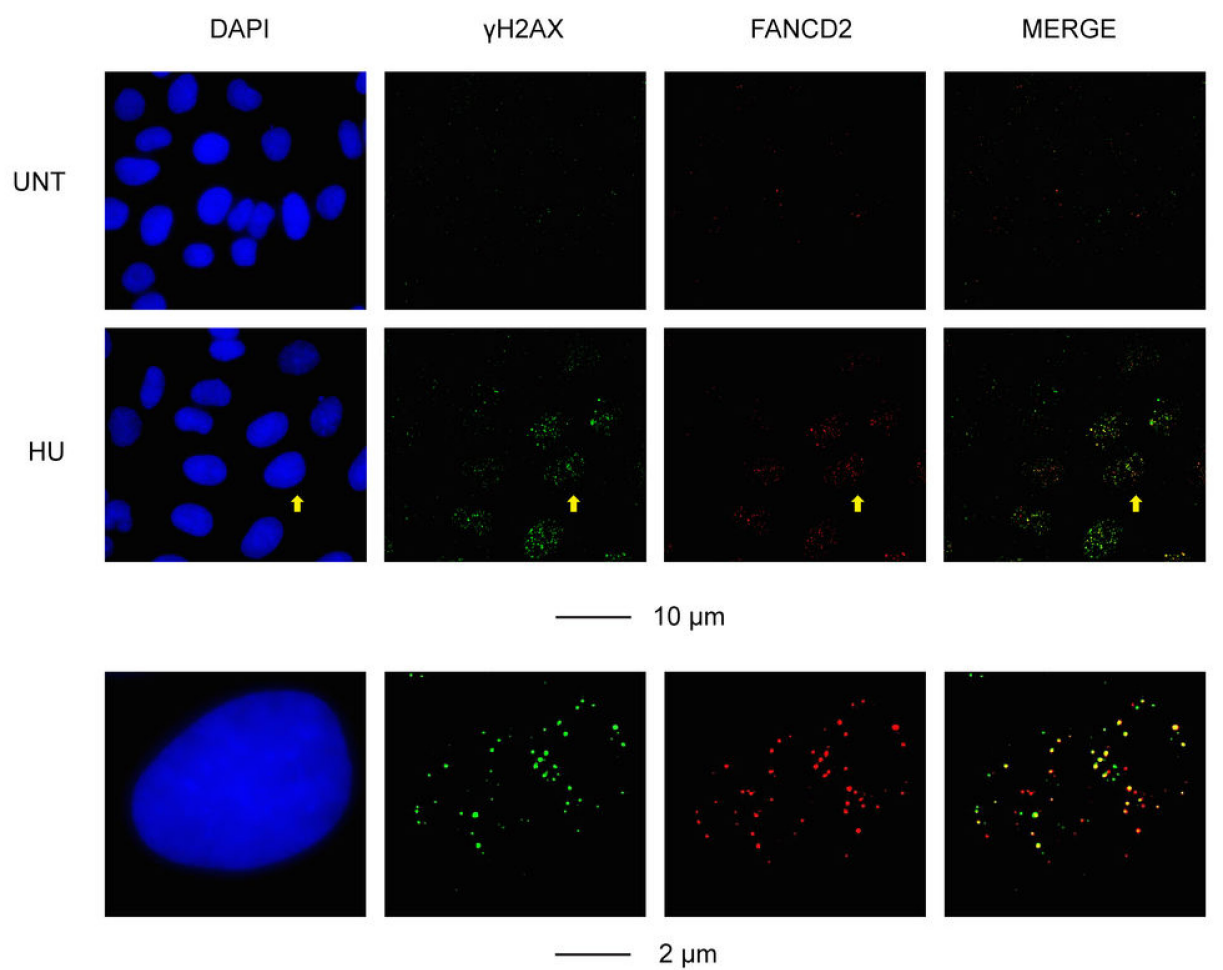
Representative images of the cellular response to RS (induced by a low dose of hydroxyurea; HU) in U2OS cells, as determined by IF staining for γH2AX and FANCD2 A selected cell is defined by the yellow arrow, and is enlarged in the bottom panel. Scale bars are indicated.

Because RS is a common feature of most cancer cells, it is likely that RS exists in one form or another in a very wide range of cancer types. A corollary to this, therefore, is that the proteins involved in RS response pathways are strong candidates to be used as a biomarker of the degree of RS, malignant potential or response to treatment in many cancer types. As a first step in testing this hypothesis, we carried out a pilot study to analyze the expression of five proteins (Ki-67, Cyclin E, POLD3, γH2AX and FANCD2) on a collection of 32 tumor samples from a representative range of cancer types (colon, lung, breast or stomach). These samples were chosen also because they represent a similar pathology grade (Table 1). The aim of this pilot study was to validate whether some of these markers are expressed in a consistent manner of tumors of similar histological grade regardless of their tissue origin or clinical stage. We optimized conditions for detection of the above proteins on paraffin-embedded tissue sections using immunohistochemical (IHC) staining method, as most of these proteins have only been detected in cultured cells using immunofluorescence (Figure 2). Our results provide some expected and some very unexpected patterns of expression (Table 1). For example, the expression patterns of POLD3 and Cyclin E are strikingly similar in all of the samples (Table 2). These data correlate with the fact that POLD3 plays an important role in DNA replication and Cyclin E promotes S-phase entry. It might be expected, therefore, that the expression pattern of Cyclin E and POLD3 would correlate well with that of Ki-67, an established proliferation marker used in pathology studies worldwide. However, they did not. Another curious finding was that the expression of γH2AX and FANCD2 rarely correlated with each other, despite the fact that *in vitro* studies using cell lines have indicated that γH2AX and FANCD2 activation are associated with RS, and commonly used as surrogate markers of RS arising at CFSs (Figure 3). Moreover, POLD3 plays a key role in counteracting RS at CFSs, and yet its expression did not correlate with either that of γH2AX or FANCD2. Overall, therefore, it is clear that the widely used RS markers in cell line studies need very careful evaluation in clinical biopsy samples before being considered serious contenders as useful biomarkers. Finally, it is worth noting that there was no obvious difference in the expression pattern of the proteins tested in the samples from different tumor types, which is consistent with the notion that RS is a common feature of a wide range of cancer types.

**Table 1:** A summary of the expression of Ki-67, Cyclin E, POLD3, γH2AX, and FANCD2 in 32 FFPE specimens from colon, lung, breast, and stomach cancer patients by IHC analysis

Case Number	Sex	Age	Tumor location	Macroscopic type	Histologic type	Degree of differentiation	Clinical stage	Ki-67	Cyclin E	POLD3	γH2AX	FANCD2
1	F	50	Colon, Right-side	Ulcerative type	adenocarcinoma	Moderate	IIA	++	++	++	-	++
2	F	58	Colon, Right-side	Ulcerative type	adenocarcinoma	Moderate	IIA	++	++	++	-	++
3	F	68	Colon, Right-side	Ulcerative type	adenocarcinoma	Moderate	IIA	-	++	++	++	++
4	F	69	Colon, Right-side	Ulcerative type	adenocarcinoma	Moderate	IIA	-	++	++	-	-
5	M	28	Colon, Transverse+sigmoid	Ulcerative type	adenocarcinoma	Moderate	IIIB	+	++	++	-	++
6	M	50	Colon, Rectum	Ulcerative type	adenocarcinoma	Moderate	IIA	+	++	++	+	++
7	F	76	Colon, Right-side	Ulcerative type	adenocarcinoma	Moderate	IIA	-	++	++	-	+
8	M	71	Colon, Sigmoid colon	Ulcerative type	adenocarcinoma	Moderate	IIA	++	++	++	++	+
9	F	76	Colon, Right-side	Ulcerative type	adenocarcinoma	Moderate	IIA	+	++	++	+	++
10	F	62	Colon, Ileocecus	Ulcerative type	adenocarcinoma	Moderate	IIIC	++	++	++	+	-
11	M	51	Lung, superior lobe of right lung	peripheral type	squamous carcinoma	Moderate	IA	++	+	++	+	+
12	M	66	Lung, superior lobe of left lung	peripheral type	squamous carcinoma	Moderate	IB	++	++	+	-	+
13	M	51	Lung, superior lobe of right lung	central type	squamous carcinoma	Moderate	IIIA	-	+	-	-	-
14	M	61	Lung, inferior lobe of right lung	central type	squamous carcinoma	Moderate	IA	++	-	++	-	++
15	M	57	Lung, superior lobe of left lung	peripheral type	adenosquamous carcinoma	Moderate	IB	-	+	++	+	-
16	M	68	Lung, superior lobe of right lung	peripheral type	adenocarcinoma	Moderate	IA	-	-	+	+	-
17	F	56	Lung, right lung	peripheral type	adenocarcinoma	Moderate	IA	-	++	-	++	+
18	M	52	Lung, superior lobe of left lung	central type	squamous carcinoma	Moderate-low	IIB	+	++	++	+	-
19	F	57	Breast, right side	/	infiltrating lobular carcinoma	Moderate	IA	-	+	++	-	-
20	F	45	Breast, left side	/	infiltrating ductal carcinoma	Moderate	IIA	-	++	++	+	-
21	F	46	Breast, left side	/	infiltrating ductal carcinoma	Moderate	IIA	-	+	++	-	-
22	F	50	Breast, right side	/	infiltrating ductal carcinoma	Moderate	IA	-	+	++	-	-
23	F	56	Breast, left side	/	infiltrating ductal carcinoma	Moderate	IIA	-	+	+	-	++
24	F	47	Breast, right side	/	introductal carcinoma	Moderate	0	-	+	++	-	+
25	F	60	Breast, left side	/	infiltrating ductal carcinoma	Moderate	IA	-	+	++	-	-
26	F	51	Breast, left side	/	infiltrating ductal carcinoma	Moderate	IIA	-	+	+	-	-
27	F	36	Breast, right side	/	infiltrating ductal carcinoma	Moderate	IIIA	++	-	++	-	+
28	F	56	Stomach	Ulcerative type	adenocarcinoma	Moderate	IIA	-	++	++	-	-
29	M	47	Stomach	Ulcerative type	adenocarcinoma	Moderate	IB	-	+	++	+	+
30	F	42	Stomach	Ulcerative type	adenocarcinoma	Moderate	IIA	-	+	++	+	++
31	F	67	Stomach	Ulcerative type	adenocarcinoma	Moderate	IB	-	++	++	-	+
32	M	63	Stomach	Ulcerative type	adenocarcinoma	Moderate	IB	-	++	++	+	++

F: female. M: male. ++: strong expression. +: moderate expression. -: negative or very weak expression.

**Table 2:** Correlation analysis of the expression of Ki-67, Cyclin E, POLD3, γH2AX, and FANCD2 in 32 FFPE specimens from colon, lung, breast, and stomach cancer patients

	Ki-67	Cyclin E	POLD3	γH2AX	FANCD2
**Ki-67**	1	0.34375	0.4375	0.5625	0.65625
**Cyclin E**		1	**0.84375**	0.46875	0.5625
**POLD3**			1	0.4375	0.59375
**γH2AX**				1	0.53125
**FANCD2**					1

The expression scores for each protein in each sample were classified into two categories: strong/moderate expression (++, or +) or absent/very weak expression (-). The two categories were then subjected to Jaccard similarity coefficient analysis for each protein. Jaccard index operates on a scale of 0–1, with the higher the score denoting cases where the expression pattern between the two proteins examined is more similar. The Protein names are in bold and the highest Jaccard index number is highlighted in bold.

## DISCUSSION

**Figure 4:**
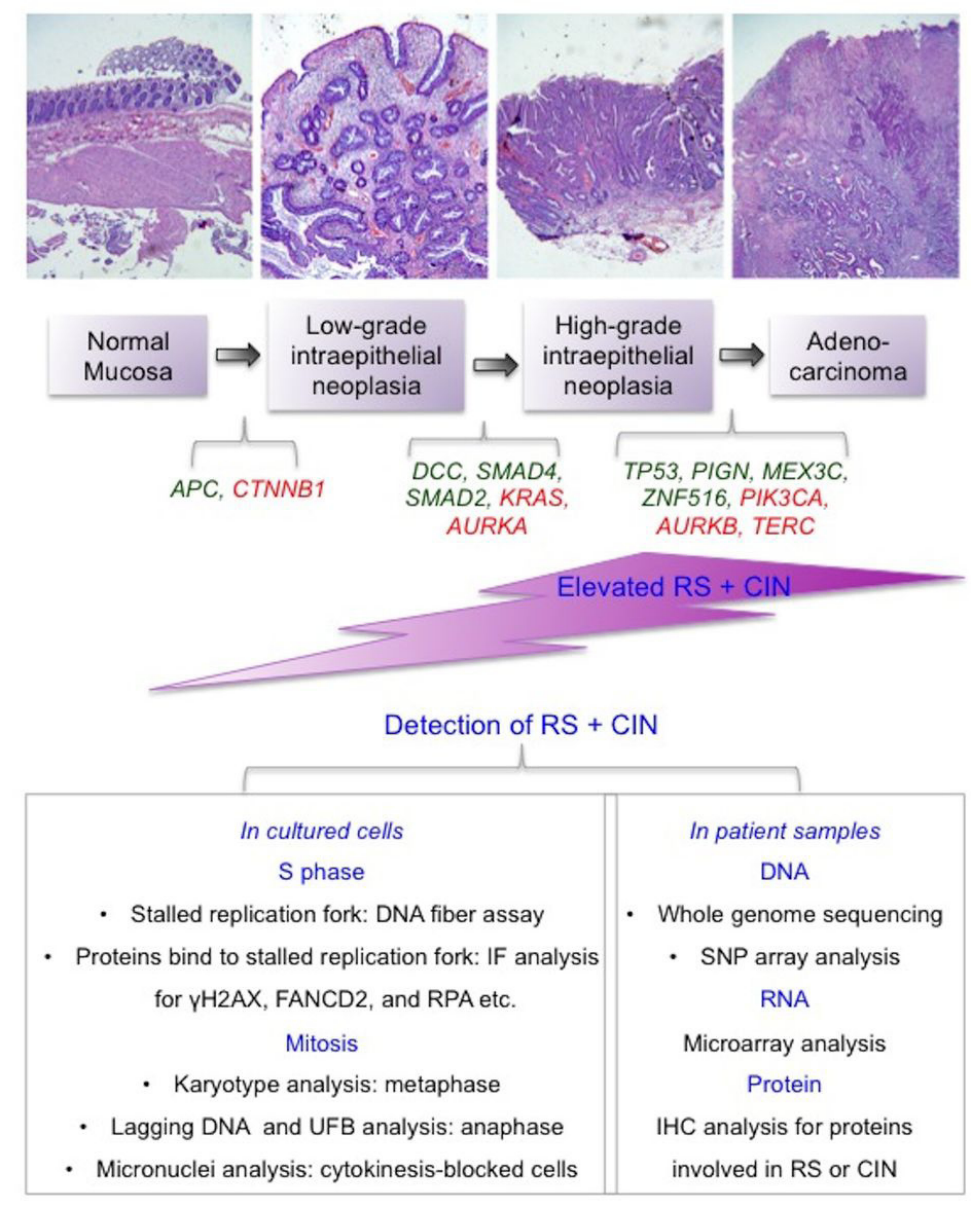
A summary of the underlying mechanisms and detection of RS and CIN in cancer samples Top panel: An illustration of increasing levels of RS and CIN during multi-step carcinogenesis using colorectal cancer as an example. During this process, multiple tumor suppressor genes (in green below the diagram) lose their function in the maintenance of genome stability, while multiple oncogenes (in red below the diagram) become activated and stimulate cells to proliferate. The RS and CIN thus formed become mutually reinforcing events that allow the cancer cells to ‘evolve’ and develop metastatic potential and drug resistance. Lower panel: A summary of the methods that can be applied to the detection of RS and CIN in laboratory or clinic settings. RS: replication stress. CIN: chromosome instability. UFB: ultra-fine anaphase DNA bridge. IF: immunofluorescence. IHC: Immunohistochemistry.

In recent years, there has been considerable progress in understanding the molecular events occurring during tumorigenesis. It is clear that abnormalities during DNA replication and the accompanying RS are common features of cancer cells. RS could be one of the most important drivers of genome instability, which in turn would permit cancer cells to develop phenotypic heterogeneity, such as an ability to metastasize or to acquire resistance to chemotherapy. Cancer cells have clearly evolved mechanisms to cope with persistent inherent RS. At the present time, we are in need a biomarker that can reliably detect RS in biopsies or reveal the cellular responses to RS that exist in cancers. Because of the routine use of cancer tissue pathology in clinics worldwide, it is important that these markers are robust enough to be studied using IHC based methods (Figure 4). Our pilot study indicates that it is possible to develop this kind of biomarker by testing various components in the RS pathways on both cancer cells in the laboratory and biopsies from cancer patients. Similar analysis should be performed in a larger collection of specimens with more comprehensive analysis relevant to the pathological and clinical characteristics of the tumors. Nevertheless, we hope this article, together with its pilot study, will facilitate future studies in this direction. The outcome of any biomarker analysis in the RS pathway would allow us to define whether the expression of proteins involved in the RS pathway could be a suitable predictor of the prognosis of patients treated with existing chemotherapy, or whether it could be used to stratify patients for treatment with one of the RS inhibitors that is being developed to target the RS pathway.

## MATERIALS AND METHODS

### Cell lines and culture conditions

Osteosarcoma cells (U2OS, obtained from ATCC) were maintained in Dulbecco's modified Eagle's medium (DMEM, Thermo Fisher Scientific) supplemented with 10% fetal bovine serum (FBS) and antibiotics. GM06865 lymphocytes (obtained from Coriell Biorepository) were maintained in RPMI 1640 medium (Thermo Fisher Scientific) supplemented with 15% FBS and antibiotics. Both types of cells were cultured at 37°C in an atmosphere of 5% CO2. For replication stress analysis, asynchronously growing cells were seeded onto glass coverslips (Sigma-Aldrich) and were treated with 0.2 mM hydroxyurea (HU; Sigma-Aldrich) for 16 hours. For karyotype analysis, asynchronously growing cells were treated with 0.1 µg/ml colcemid (Karyomax, Thermo Fisher Scientific) for 5 hours before being harvested.

### Immunofluorescence (IF) analysis

Cells fixed on glass cover slips were blocked with 3% BSA (Sigma-Aldrich) for 1 hour at room temperature and incubated with primary antibodies at 4°C overnight, followed by three washes (30 minutes each) using PBST (PBS with 0.2% Triton-X 100, Sigma-Aldrich). Slides were then incubated with secondary antibodies for 1 hour at room temperature, followed by three washes (30 minutes each) using PBST. Air-dried coverslips were mounted using Vectashield mounting medium with DAPI (Vector Laboratories), and were analyzed with a Retiga6000 camera connected to an Olympus BX63 microscope. The origins and dilutions of primary and secondary antibodies were: anti-FANCD2 (1:400, NB100-182; Novus), anti-γH2AX (1:400, JBW-301; Millipore), Alexa Fluor 568 goat anti-rabbit IgG (1:500, A-1101; Invitrogen) and Alexa Fluor 488 goat anti-mouse IgG (1:500, A-110291; Invitrogen).

### Karyotype analysis

The karyotype of metaphase cells was obtained by standard G-banding and analyzed using a Leica light microscope equipped with CytoVision software.

### Clinical specimens

Archival blocks of formalin-fixed, paraffin-embedded (FFPE) tumor, neoplasia or normal specimens from the colon (n=13), lung (n=8), breast (n=9), or stomach (n=5) were obtained in accordance with local Research Ethics guidelines. All of the specimens were chosen according to their histological classification and the degree of cell differentiation. Information concerning the tumor specimens chosen for immunohistochemical (IHC) staining is shown in Table 1.

### IHC staining and evaluation

Sections (5 µm) were cut onto aminopropyltriethoxysilane (APES)-coated slides from each specimen. In each case, one of the sections was subjected to standard hematoxylin and eosin (H&E) staining to confirm histological diagnosis, while the rest were subjected to IHC analysis using a SP-9000, SPlink Detection Kit following manufacturer's instructions (Zhong Shan -Golden Bridge Biological Technology Co. Ltd., Beijing, China). The origins and the dilutions of the primary antibodies used are: anti-gamma H2AX (polyclonal, Abcam, ab2893, 1:400), anti-POLD3(monoclonal, Abcam, ab182564, 1:70), anti-FANCD2 (monoclonal, Abcam, ab108928, 1:80), anti-CyclinE (polyclonal, BIOSS, Bs-8929R, 1:150), and anti-Ki67 (monoclonal, Gene Tex, GTX83375, 1:400). All of the primary antibodies were incubated with pre-blocked slides at 4 °C overnight. The secondary antibodies were goat anti-mouse or goat anti-rabbit HRP for 30 minutes. 3,3’-diaminobenzidine (DAB) was used as the chromogen. Negative controls were preformed by omitting the primary antibody. The images were captured using an Olympus DP73 camera connected to an Olympus BX53 microscope. Staining results were assessed independently by three pathologists on coded samples.

The expression level of the protein analyzed was scored as described in [59]. In brief, the intensity of IHC staining was scored from 0 to 4 as: 0, negative; 1, weak; 2, moderate; and 3, strong. The proportion of the cells stained positively was scored from 0 to 4 as: 0, less than 5%; 1, 5-25%; 2, 26-50%; 3, 51-75; and 4, over 76%. A final ‘expression score’ for each antibody in each specimen was achieved by multiplying the scores of intensity and proportion of the positive cells. Scores of 9-12 were defined as “strong expression”, scores of 5-8 were defined as “reduced expression”, and scores of 0-4 were defined as “negative or markedly reduced expression”.
